# A Manual of the Practice of Medicine

**Published:** 1893-04

**Authors:** 


A Manual of the Practice of Medicine. By A. A. Stevens, A. M., M. D., Instructor of Physical Diagnosis in the University of Pennsylvania, and Demonstrator of Pathology in the Woman's Medical College, Philadelphia. Illustrated. Philadelphia: W. B. Saunders, 913 Walnut Street. Price, $2.50.

This hand-book of the Practice of Medicine was written for the use of students especially, with, as the author expresses himself, "the hope that it may serve as an outline to be enlarged upon by diligent attendance upon lectures and critical observations at the death-bed." The book is convenient in form, well printed, and contains valuable material from works of the most learned professors and medical teachers of our day. It is to be recommended to students who need such a useful and convenient work. It contains five hundred pages, and the descriptions of the various diseases are condensed into as small a space as possible, so that the physician who is pressed for time may find what he wants quickly without having to wade through an elaborate description. The arrangement of the book is according to the natural system: diseases of the digestive system; diseases of the kidneys; diseases of the blood; diseases of the respiratory system, and the like.

